# Usability and Feature Evaluation of the Amazfit Bips Smart Watch in the Precision START Lab

**DOI:** 10.1093/geroni/igaa057.634

**Published:** 2020-12-16

**Authors:** Jo-Ana Chase, Chelsea Howland, Malaika Gallimore, Blaine Reeder

**Affiliations:** 1 University of Missouri, Columbia, Columbia, Missouri, United States; 2 University of Missouri - Columbia, Sinclair School of Nursing, Columbia, Missouri, United States; 3 University of Missouri, Columbia, Missouri, United States

## Abstract

Interventions utilizing consumer-grade wearable and mobile devices may support older adult health and wellness. However, rapid technology change and short industry product release cycles limit timely incorporation of these devices. We developed a novel, multi-stage process to rapidly move from within-team evaluations to lab- and field-based participants studies based on small-sample technology testing methods from Human-Computer Interaction. We present findings from a first-stage evaluation of the Amazfit Bips smart watch for potential use in studies with older adults as part of the methodology validation. A four-person research team conducted evaluations using: 1) a wearables framework for user experience and feature availability; and 2) the System Usability Scale (SUS). Evaluators wore the watch seven days straight from the box. User experience checklists indicated high usability. However, corresponding comments identified challenges with downloading the mobile app, pairing the watch and phone, navigating watch and mobile interfaces, and privacy controls. Average SUS score was 65.6 indicating marginal usability (C grade). While meeting study goals, divergence in usability perceptions suggest the process could be improved by completing each set of instruments separately for the watch and mobile app rather than all at once. Given failures in pairing, app navigation challenges, and small screen size, the Amazfit Bips may be best suited for studies among older adults with a high degree of technical proficiency. For those with little technical experience or high disease burden, training materials and dedicated training with support may be required. Future steps are lab- and field-based tests with older adult participants.

